# Bibliometric and visual analysis of transcranial direct current stimulation in the web of science database from 2000 to 2022 *via* CiteSpace

**DOI:** 10.3389/fnhum.2022.1049572

**Published:** 2022-12-01

**Authors:** Weiming Sun, JingJing Song, Xiangli Dong, Xizhen Kang, Binjun He, Wentao Zhao, Zhaoting Li, Zhen Feng, Xiuping Chen

**Affiliations:** ^1^Department of Rehabilitation Medicine, The First Affiliated Hospital of Nanchang University, Nanchang, China; ^2^Jiangxi Medical College, Nanchang University, Nanchang, China; ^3^Department of Psychosomatic Medicine, The Second Affiliated Hospital of Nanchang University, Nanchang, China; ^4^School of Life Science, Nanchang University, Nanchang, China; ^5^The Third Clinical Department, China Medical University, Shenyang, China

**Keywords:** transcranial direct current stimulation, bibliometric analysis, CiteSpace, research trends, web of science transcranial direct current stimulation, web of science

## Abstract

**Objective:**

This study aimed to evaluate the current research hotspots and development tendency of Transcranial Direct Current Stimulation (tDCS) in the field of neurobiology from a bibliometric perspective by providing visualized information to scientists and clinicians.

**Materials and methods:**

Publications related to tDCS published between 2000 and 2022 were retrieved from the Web of Science Core Collection (WOSCC) on May 5, 2022. Bibliometric features including the number of publications and citations, citation frequency, H-index, journal impact factors, and journal citation reports were summarized using Microsoft Office Excel. Co-authorship, citation, co-citation, and co-occurrence analyses among countries, institutions, authors, co-authors, journals, publications, references, and keywords were analyzed and visualized using CiteSpace (version 6.1.R3).

**Results:**

A total of 4,756 publications on tDCS fulfilled the criteria we designed and then were extracted from the WOSCC. The United States (1,190 publications, 25.02%) and Harvard University (185 publications, 3.89%) were the leading contributors among all the countries and institutions, respectively. NITSCHE MA and FREGNI F, two key researchers, have made great achievements in tDCS. *Brain Stimulation* (306 publications) had the highest number of publications relevant to tDCS and the highest number of citations (4,042 times). In terms of potential hotspots, we observed through reference co-citation analysis timeline viewer related to tDCS that “depression”#0, “Sensorimotor network”#10, “working memory”#11, and “Transcranial magnetic stimulation”#9 might be the future research hotspots, while keywords with the strong burst and still ongoing were “intensity” (2018–2022), “impairment” (2020–2022), “efficacy” (2020–2022), and “guideline” (2020–2022).

**Conclusion:**

This was the first-ever study of peer-reviewed publications relative to tDCS using several scientometric and visual analytic methods to quantitatively and qualitatively reveal the current research status and trends in the field of tDCS. Through the bibliometric method, we gained an in-depth understanding of the current research status and development trend on tDCS. Our research and analysis results might provide some practical sources for academic scholars and clinicians.

## Introduction

Non-invasive brain stimulation (NIBS) that consists of transcranial magnetic stimulation (TMS) and transcranial electrical stimulation (TES) has developed rapidly in the past 30 years and has been widely used in behavioral and cognitive science (Weng et al., 2020). The former stimulates nerves in the brain by generating a localized magnetic induction current through a coil, while the latter delivers a weak current (usually between 0.4 and 2.0 mA) to the head through electrodes on the scalp, directly stimulating a localized brain region (Nasseri et al., 2015).

As a type of TES, transcranial direct current stimulation (tDCS) is a promising method for altering the function of neural systems, cognition, and behavior (Chase et al., 2020). It is a neuromodulation technique that non-invasively alters cortical excitability *via* weak polarizing currents between two electrodes placed on the scalp. Since tDCS is comparably easy to handle, cheap to use, and relatively well-tolerated, it has gained increasing interest in recent years. Several clinical studies have been performed in populations including patients with major depressive disorder followed by schizophrenia and substance use disorders, with heterogeneous results concerning efficacy (Herrera-Melendez et al., 2020).

Development trend analysis of disciplines (referring to all fields of science) can make intelligence personnel, scientific research managers, and decision-makers a comprehensive understanding of the relevant research in a relatively short period. Moreover, the quality of publications is advantageous for researchers to increase the level of scientific research, and it is also conducive for scientific administrators and policymakers to make decisions and adjust the direction and layout of research (Weng et al., 2020). Bibliometrics is the basis of network info metrics, while bibliometric analysis can quantify the impact of individual research results and the literature development of specific subjects, and evaluate the tendencies of scientific research and info metrics (Chen, 2004). The bibliometric methods discover the knowledge relationship between publications by screening and analyzing a massive amount of data, therefore, mining out the potential knowledge value (Yu et al., 2019). Bibliometrics is not only the interdisciplinary science of quantitative analysis of all knowledge carriers by mathematical and statistical methods but also a comprehensive knowledge system that integrates mathematics, statistics, and philology as a whole and pays attention to quantitative analysis. The most obvious advantage is that it enables researchers to explore the specific research field by analyzing the citations, co-citations, distribution, and term frequency. In other words, it is possible to investigate the inner publication structure and citation landscape of a particular field of study (Yu et al., 2018). With the continued evolution of bibliometric methodology, the development and potential direction of future research can be predicted through bibliometric analysis for it can provide a roadmap for further research. Hitherto, bibliometric studies have been widely used in various areas, such as medical big data, pain, cognitive function, and neuroimaging in recent years (Weng et al., 2020). Bibliometric analysis has been made using bibliometric software, including CiteSpace, VOSviewer, bibExcel, Science of Science (SCI2), and HistCite, but CiteSpace is one of the most popular.

A considerable number of scholars and academic journals have been focusing on research related to tDCS over the last 20 years. However, studies on trends of tDCS through a bibliometric analysis were rare. Based on documents relevant to tDCS from 2000 to 2022, we used CiteSpace to identify the publication patterns and emerging trends of this technique and gain new insights in order to guide future research and application.

## Materials and methods

### Data source

With the availability of bibliometric indicators and more than 12,000 significant high-quality journals from nations throughout the world, Web of Science (WoS), one of the most comprehensive, systematic, and authoritative databases, is widely used for bibliometrics analysis and visualization of scientific literature (Wu et al., 2021c), and Dr. Chaomi Chen originally developed the dataset extracted from WoS as an evaluation testbed for the Citespace system. Significantly, data downloaded from the WoS could directly provide reference files that satisfy the specific format requirements set by the bibliometric software CiteSpace. Otherwise, if data were collected from other databases, an additional process for file format conversion should be required (Synnestvedt et al., 2005). In addition, the accuracy, reliability, and representativeness of a certain dataset rely on the authority of the database, so the Science Citation Index Expanded (SCI-Expanded) of Web of Science Core Collection (WoSCC) database was chosen as the data source in this study. Publications extracted in this study were all from the WOSCC published from January 2000, 2022, to May 5, 2022 (approximately 22 years) as the input data for CiteSpace to identify key points and analyze dynamic trends in scientific research on tDCS. The majority of publications on tDCS are included in the WoS online database, which allowed us to extract the relevant articles using an appropriate retrieval strategy.

### Retrieval strategies

**FIGURE 1:**
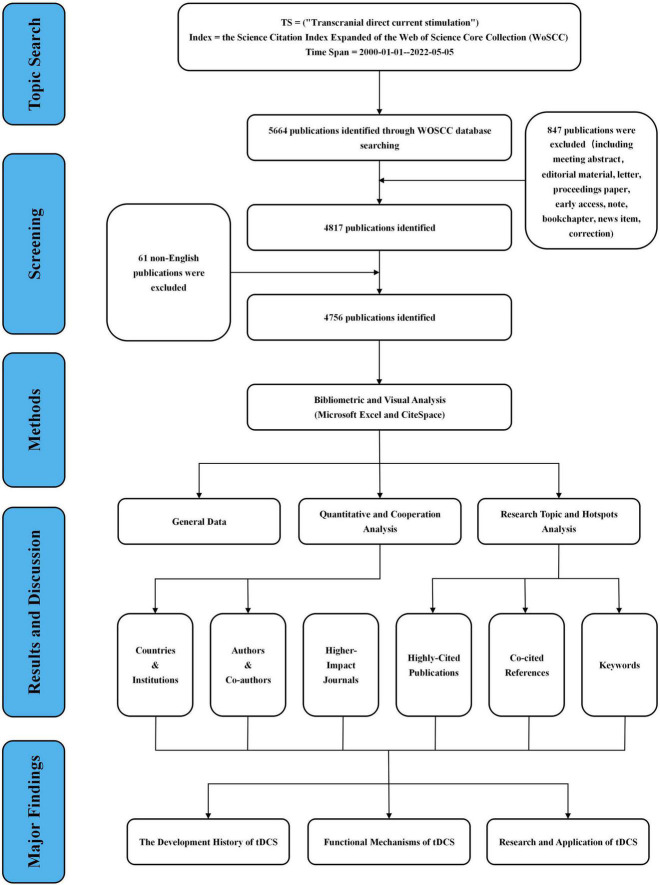
Flowchart of literature screening included in this study.

Publications were retrieved *via* the topic search of the Science Citation Index Expanded (SCI-EXPANDED) of the WoS database. As shown in Figure 1, the following search terms were used: topic = (“Transcranial Direct Current Stimulation” or “tDCS”), index = SCI-EXPANDED, time span = 2000–2022, and Language = English. Only original articles and reviews were included. Other document types, such as meeting abstracts and letters, were excluded. Finally, there remained 4,756 publications. To avoid bias incurred by frequent database renewal, all literature searches and data downloads were accomplished on May 5, 2022.

### Analysis tool

CiteSpace is a tool for visualization analysis that concentrates on examining patterns and dynamic changes in publications of scientific research and tries to identify key points in a certain field (Chen, 2006). In this study, it was used to evaluate the collaborative relationships and co-citation between authors and journals and detect citation bursts for references.

CiteSpace, an interactive visualization tool that is great at performing bibliometric analysis, is also a Java application that combines methods of information visualization, bibliometrics, and data mining algorithms (Kim et al., 2016; Chen, 2017). CiteSpace (6.1.R3) is used to perform co-citation analysis, and it can also summarize and visualize the collaborations into a network map that consists of a series of points and lines (de Paulo et al., 2018), and a wider line indicates a stronger relationship. More importantly, CiteSpace (6.1.R3) can detect keywords and references with citation bursts. A citation burst has two characteristics, strength and duration (Chen, 2006). An item with a citation burst indicates that it gains increasing attention over a certain period of time, which is a key indicator for the assessment of emerging trends (Chen et al., 2014).

We downloaded the records retrieved from the WOSCC and then converted these data into plain text format for export which was named “download_ XXX.txt,” including complete records and references. Finally, we imported these data into CiteSpace (6.1.R3) for bibliometric and visualized analysis. Cluster analysis of co-occurrence keywords that reveal the main topics was performed by CiteSpace. The silhouette function is usually used to assess the clusters. Generally speaking, if the silhouette value is over 0.7, it means that the members of the cluster have high homogeneity, indicating that the clustering result is meaningful. If it is > 0.5, clustering is generally considered reasonable.

## Results and discussion

### General data

**FIGURE 2:**
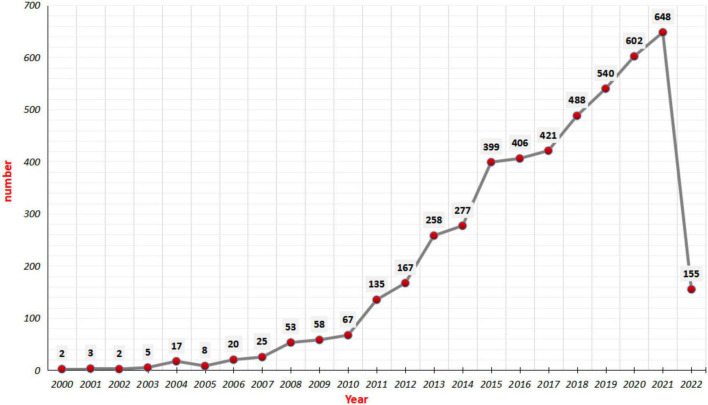
The number of publications related to transcranial direct current stimulation (tDCS) extracted from the web of science core collection (WOSCC), 2000–2022.

A total of 4,756 relevant documents (Figure 1) were extracted from the WOSCC database with no duplicate records. The number of publications and citations in each period can be a direct reflection of the development trend of scientific knowledge in a certain field. As shown in Figure 2, the number of original articles in English related to tDCS showed a roughly year-on-year increasing trend, from only two articles published in 2002 to 648 articles published in 2021. Significantly, the average growth rate of scientific publications on tDCS was 31.69% from 2000 to 2021, which indicated that research in this field is developing rapidly. In particular, the rapid growth in 2010 suggested that research related to tDCS received much more attention that year. The number of articles on tDCS published in 2021 is 648, which accounted for 13.62% of the total quantity, and 155 publications have been published from January 1, 2022, up to May 5, 2022.

### Quantitative and cooperation analysis

#### Bibliometric analysis of countries and institutions

According to the number of publications on tDCS issued by countries and institutions, the top ten countries are noted in Table 1, and we correspondingly selected the institutions with the highest number of publications in those countries. In addition, we selected the top three institutions in the top three countries by the number of publications. That is to say, the major productive co-institutes in the field of tDCS are also presented in Table 1. The H-index that is a simple and useful indicator to characterize the output of a researcher is defined as follows: whether a scientist has an index *h* means that *h* of his or her publications has at least *h* citations, and each remaining publication has fewer citations than *h*. This metric enables an assessment of the quality and quantity of publications (Hirsch, 2005). Furthermore, H-index is often used as a quantitative and qualitative measure of an academic researcher’s production, and it may also be used to describe the publications of a nation, a journal, or an institution (Engqvist and Frommen, 2008).

**TABLE 1:** The top 10 most prolific countries/regions and corresponding institutions in research of transcranial direct current stimulation (tDCS).

Ranking	Country/Region	Frequency/*N* (%)	Centrality	Institution	Frequency/*N* (%)	Centrality	Average citation	H-index
1	USA	1,190/25.02	0.49	Harvard Univ	185/3.89	0.42	47.29	121
				Harvard Med Sch	140/2.94	0.21		
				CUNY City College	97/2.04	0.07		
2	Germany	744/15.64	0.99	Univ Gottingen	135/2.84	0.47	70.75	128
				Leibniz Res Ctr Working Environm & Human Factors	74/1.56	0.28		
				Univ Med Hosp Bergmannsheil	66/1.39	0.21		
3	Italy	495/10.41	0.10	Univ Milan	34/0.71	0.22	43.56	74
				Univ Milano Bicocca	38/0.80	0.16		
				Univ Brescia	20/0.42	0.19		
4	England	438/9.21	0.34	UCL	89/1.87	0.23	49.93	78
5	Brazil	365/7.67	0.42	Univ São Paulo	158/3.32	0.75	49.26	70
6	Australia	332/6.98	0.29	Monash Univ	77/1.62	0.04	36.33	64
7	China	328/6.90	0.05	Hong Kong Polytech Univ	10/0.21	0.00	10.87	33
8	Canada	232/4.88	0.15	Univ Toronto	59/1.24	0.20	26.25	44
9	France	170/3.57	0.68	Hop Henri Mondor	6/0.13	0.06	40.15	47
10	Spain	167/3.51	1.34	Univ Autonoma Barcelona	8/0.17	0.00	39.26	42

**FIGURE 3:**
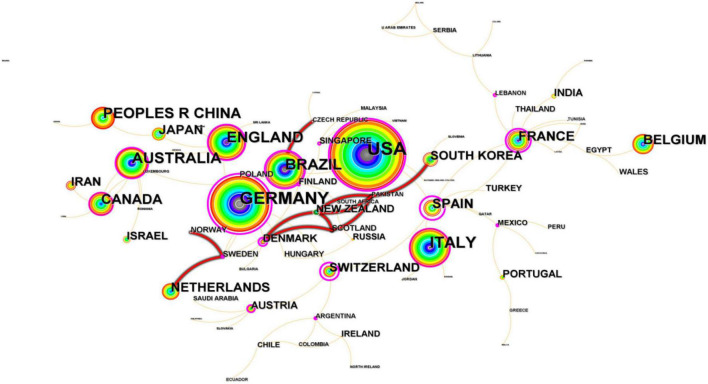
A map of some countries’ cooperative relations in research of transcranial direct current stimulation (tDCS), 2000–2022.

**FIGURE 4:**
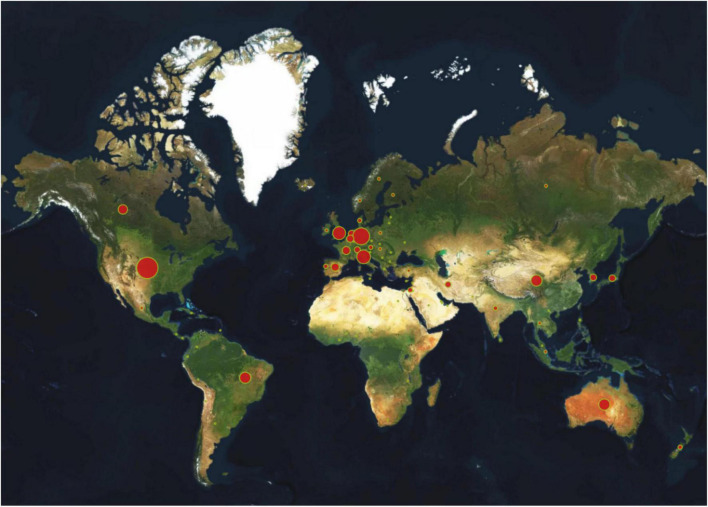
A world map depicting the contribution of each country based on publication counts.

As presented in Table 1, the USA (1,190, 25.02%) ranked first with absolute contribution and relatively high influence, reflecting its dominant position in the field of tDCS, followed by Germany (744, 15.64%) and Italy (495, 10.41%). Significantly, Germany ranked second in the number of publications, while it ranked first in terms of the average citation of each article and H-index (70.75, 128). It is also evident that China (328 publications, Centrality = 0.05) and Canada (232 publications, Centrality = 0.15) occupied the seventh and eighth positions in the number of publications, but as far as the centrality is concerned, both the average citation of each article and H-index were relatively much lower than those of some countries in the USA and Western Europe. As a result, there is still a need for improving the quality of publications except for the increase in quantity in these two countries. Furthermore, it is widely acknowledged that international and interorganizational collaboration is a significant strategy to increase the quality and productivity of research in our increasingly interdependent and globalized society. In the CiteSpace Atlas, the higher the number of original articles published is, the larger the size of the circle, and the line thickness between the two countries indicates the strength of cooperation. Additionally, the larger the scale of cooperation is, the thicker the connection line. A map of some countries’ cooperative relations in research of tDCS is presented in Figure 3, and the USA (1,190, 25.02%) had the largest circle, which indicated that the number of tDCS-related articles published in the USA was the highest. Center on Germany (Centrality = 0.99), France (Centrality = 0.68), the USA (Centrality = 0.49), etc., countries around them were closely connected. The lines between the nodes with different colors represent different years of cooperation between countries or institutions. According to the color gradient, the bold red lines showed the recent partnership between countries in Figure 3. Figure 4 displayed a globe map that showed the contributions made by nations. We can observe clearly from the color-coded gradient that scholars from places such as North America, South America, Western Europe, and Eastern Asia published the great majority of literature. In addition, collaborations between countries might be found all throughout high-income nations such as those in North America and Western Europe. A lack of academic interaction between Asian nations and institutions was evident, since nations and institutions in these regions did not form a cooperation network, despite the fact that several Asian countries have made significant contributions to tDCS.

**FIGURE 5:**
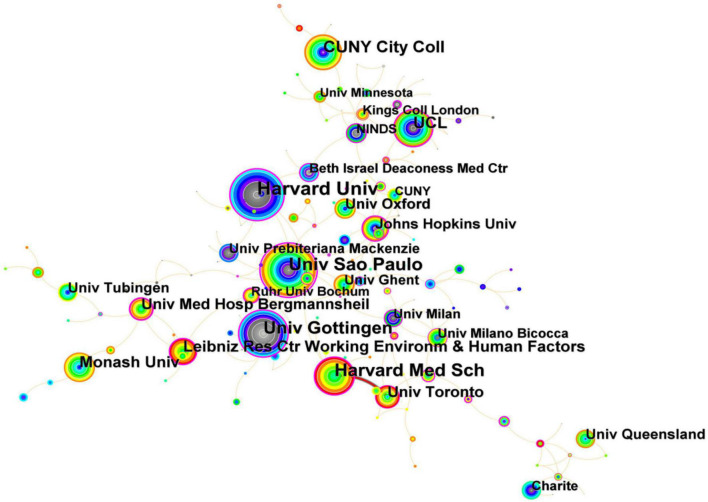
Map of top institutional relations related to transcranial direct current stimulation (tDCS), 2000–2022.

As for the analysis of institutions, it was roughly estimated that more than 164 institutions have made contributions to the field of tDCS. Especially, Harvard University was the foremost productive and influential institute in this field, with a total number of 185 publications, followed by University of São Paulo (158 publications), Harvard Medical School (140 publications), and University of Göttingen (135 publications), while a total number of articles published from the remaining institutions was less than 578. In general, institutions with strong scientific research strength were mainly distributed in higher educational research institutions. Significantly, the articles published by University of São Paulo accounted for 49.26% of entire Brazil in terms of the number of publications, while the top three institutes in the USA (Harvard University, Harvard Med Sch, CUNY City College) published 422 papers, which accounted for 47.29%. As shown in Figure 5, Harvard University and University of São Paulo had a strong cooperative relationship, and University of São Paulo and Leibniz Res Ctr Working Environm & Human Factors were active in research in 2022 (Node in red represents that it has cooperation in 2022).

“Centrality” in CiteSpace refers to intermediary centrality, which is an indicator to measure the importance of nodes in the network (Wang et al., 2020). We can use this index to discover and measure the importance of literature, and then CiteSpace applies a purple circle to highlight such literature (or authors, journals and institutions, etc., and node-intermediary centrality with purple is not less than 0.1). The greater the proportion of the purple circle to nodes is, the higher the centrality. Significantly, a country or an institution with high mediational centrality is often the key junction connecting two different fields, which is also called a turning point in CiteSpace. If the centrality of a node exceeds 0.1, it indicates that the node is a central node that is relatively important and has great influence in the field. Spain (167 publications) ranked 10th in terms of the number of articles published on tDCS, while it was with the highest centrality (1.34) among the top ten countries. Economic strength is an important factor affecting scientific output, and it is worth mentioning that eight of these countries among the top ten countries were developed countries and only two (i.e., China and Brazil) were developing countries. From this point of view, there was still a wide gap between developed countries and developing countries in this field. The centrality of University of São Paulo reached 0.75, which meant that it was the most important hub, followed by University of Göttingen (centrality = 0.47) and Harvard University (centrality = 0.42). Shanghai Jiao Tong University, University of Ghent, University of Calgary, Max Planck Institute for Human Cognitive & Brain Sciences, Center for Addiction & Mental Health, and University Leipzig have been active since 2021.

#### Bibliometric analysis of authors and co-authors

Hitherto, more than 189 scholars participated in research relevant to tDCS and accomplished more than 4,756 publications. The researchers’ effort and contribution to a certain field may be represented by the number of scientific publications written by them (Wu et al., 2021a). From Table 2, we learned that the top 10 authors contributed 999 papers (21.01%) to research on tDCS. From the perspective of publication count, the most prolific author was NITSCHE MA (206 publications) from University of Gottingen, and an article titled “Transcranial direct current stimulation: State of the art 2008” published by him along with other authors in 2008 whose cited frequency ranked second (1,891 times) gained much attention in the field of tDCS (Table 4). The next highest was FREGNI F (202 publications) from Harvard Medical School. Their study provided guidance on how to perform tDCS safely and effectively, and stimulus packages were stratified to improve the comparability of new findings (Nitsche et al., 2008). BIKSON M (125 publications) from City College of New York (CUNY) ranked third, while he collaborated with NITSCHE MA and FREGNI F several times and published some articles together.

**TABLE 2:** The top 10 most productive authors and the top 10 co-cited authors in research of transcranial direct current stimulation (tDCS).

Ranking	Author	Count	H-index	Co-cited author	Count	H-index
1	Nitsche MA	206	96	Nitsche MA	2,730	96
2	Fregni F	202	91	Fregni F	1,327	91
3	Bikson M	125	67	Brunoni AR	1,088	51
4	Brunoni AR	104	51	Boggio PS	1,033	59
5	Paulus W	103	114	Antal A	1,028	63
6	Pascual-Leone A	73	145	Stagg CJ	1,011	33
7	Boggio PS	61	59	Bikson M	724	67
8	Antal A	50	63	Gandiga PC	724	3
9	Loo CK	43	26	Liebetanz D	615	39
10	Jaberzadeh S	32	28	Lefaucheur JP	594	64

**TABLE 3:** The top 20 journals and the top 20 co-cited journals in research of transcranial direct current stimulation (tDCS).

Ranking	Journal	Output	IF	JCR	Co-cited journal	Citation	IF	JCR
1	Brain Stimulation	306	9.184	Q1	Brain Stimulation	4,041	9.184	Q1
2	Front Hum Neurosci	210	3.473	Q2	Clinical Neurophysiology	3,708	4.861	Q2
3	PLoS One	117	3.752	Q2	Neuroimage	3,248	7.400	Q1
4	Frontiers in Neuroscience	111	5.152	Q2	J Physiol-London	3,248	6.228	Q1
5	Scientific Reports	104	4.996	Q2	J Neurosci	3,188	6.709	Q1
6	Neuropsychologia	102	3.054	Q2	Neurology	2,610	11.800	Q1
7	Neurosci Lett	108	3.197	Q3	Experimental Brain Research	2,548	2.064	Q4
8	Neuroimage	90	7.400	Q1	PLoS One	2,508	3.752	Q2
9	Brain Sciences	91	3.333	Q3	Brain	2,439	15.255	Q1
10	Clinical Neurophysiology	83	4.861	Q2	Neuropsychologia	2,390	3.054	Q2
11	Experimental Brain Research	83	2.064	Q4	Eur J Neurosci	2,180	3.698	Q3
12	Eur J Neurosci	77	3.698	Q3	J Neurophysiol	2,115	2.974	Q2
13	Frontiers in Neurology	66	4.086	Q2	Front Hum Neurosci	2,089	3.473	Q2
14	Restor Neurol Neuros	69	2.976	Q3	Neurosci Lett	2,072	3.197	Q3
15	J Neurosci	60	6.709	Q1	Cereb Cortex	2,040	4.861	Q2
16	Cortex	52	4.644	Q1	P Natl Acad Sci USA	2,008	12.779	Q1
17	Neuroscience	51	3.708	Q3	Neuron	1,836	18.688	Q1
18	Behavioural Brain Research	48	3.352	Q2	J Cognitive Neurosci	1,821	3.420	Q2
19	J Neurophysiol	47	2.974	Q2	NEUROREPORT	1,638	1.703	Q4
20	Front Aging Neurosci	48	5.702	Q1	Hum Brain Mapp	1,486	5.399	Q1

**TABLE 4:** The top 10 cited-publications in research of transcranial direct current stimulation (tDCS).

Ranking	Publication	Cited frequency	
		Average annual citation frequency	Count
1	Excitability changes induced in the human motor cortex by weak transcranial direct current stimulation Nitsche, MA and Paulus, W	144.74	3,329
2	Transcranial direct current stimulation: State of the art 2008 Nitsche, MA; Cohen, LG; (…); Pascual-Leone, A	126.07	1,891
3	Sustained excitability elevations induced by transcranial DC motor cortex stimulation in humans Nitsche, MA and Paulus, W	78.91	1,736
4	Transcranial DC stimulation (OCS): A tool for double-blind sham-controlled clinical studies in brain stimulation Gandiga, PC; Hummel, FC and Cohen, LG	66.71	1,134
5	Pharmacological modulation of cortical excitability shifts induced by transcranial direct current stimulation in humans Nitsche, MA; Fricke, K; (…); Paulus, W	47.2	944
6	Physiological Basis of Transcranial Direct Current Stimulation Stagg, CJ and Nitsche, MA	47.15	943
7	Pharmacological approach to the mechanisms of transcranial DC-stimulation-induced after-effects of human motor cortex excitability Liebetanz, D; Nitsche, MA; (…); Paulus, W	43.95	923
8	Noninvasive cortical stimulation enhances motor skill acquisition over multiple days through an effect on consolidation Reis, J; Schambra, HM; (…); Krakauer, JW	62.71	878
9	Direct Current Stimulation Promotes BDNF-Dependent Synaptic Plasticity: Potential Implications for Motor Learning Fritsch, B; Reis, J; (…); Lu, B	65	845
10	Clinical research with transcranial direct current stimulation (tDCS): Challenges and future directions Brunoni, AR; Nitsche, MA; (…); Fregni, F	73.55	809

PASCUAL-LEONE A ranked 6th in publications (73 publications), while his H-index (145) was the top one among other authors. As shown in Table 2, both NITSCHE MA (Ranking = 1, 206 publications) and PAULUS W (Ranking = 5, 103 publications) are from the same research institution, and an article published by them titled “Excitability changes induced in the human motor cortex by weak transcranial direct current stimulation” in 2000 was the highest cited frequency among other articles on tDCS up to now, in which they demonstrated the possibility of a non-invasive modulation of motor cortex excitability using the application of weak direct current through the scalp in the intact human in this publication (Nitsche and Paulus, 2000).

**FIGURE 6:**
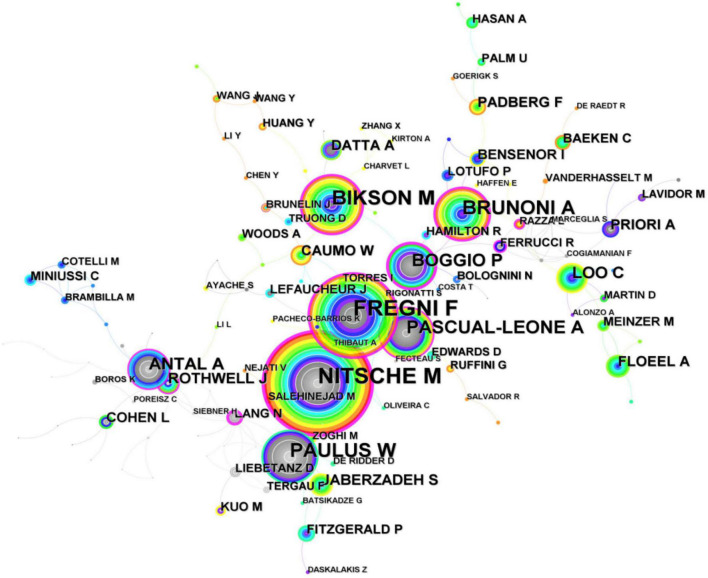
The network of authors in research of transcranial direct current stimulation (tDCS), 2000–2022.

**FIGURE 7:**
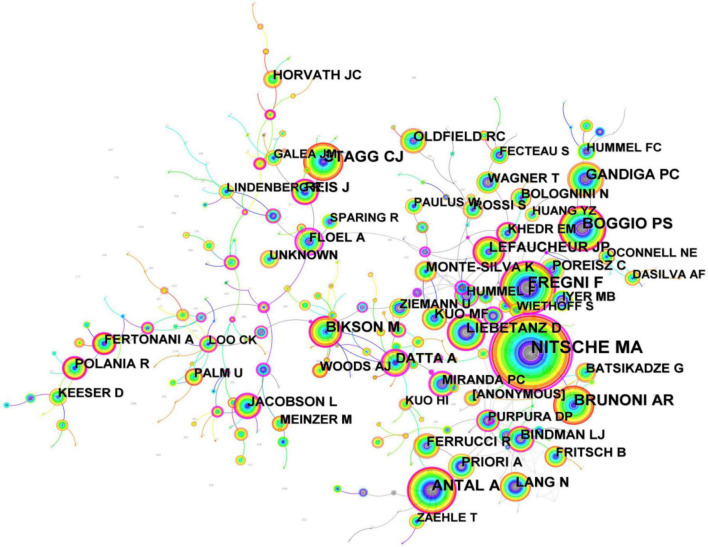
The network of cited authors in research of transcranial direct current stimulation (tDCS), 2000–2022.

Core authors lead the way in the development of science and have significant influence. Price’s theorem is often used to determine the core author, and the specific formula is as follows: *Q*0.749*C*. According to Ripps law, only when core authors’ literature account for 50% of the total number of publications can a high-yielding authors group be formed. According to the calculation, the total number of articles published on tDCS by core authors accounted for 28.62% of the total number of publications on tDCS, which showed that the core authors’ studies were more independent and less cooperative. The network of authors relevant to tDCS generated by CiteSpace is shown in Figure 6, and the thicker the line between different circles is, the more collaboration between authors. As shown in Figure 7, productive authors usually had stable collaborations with others.

Whenever two authors/publications appear together in the reference list of a third document is referred to as the co-citation connection (Wu et al., 2021b). The key authors in a field’s co-citation network are frequently revealed using the author co-citation analysis. Authors who are regularly referenced are generally regarded to have more impact than authors who are less frequently mentioned. Additionally, authors who are frequently referenced together focus on related fields of study. As presented in Table 2, in terms of co-citation count, NITSCHE MA (2,730 citations) ranked first as the author with the highest co-cited, followed by FREGNI F (1,327 citations). BRUNONI AR (1,088 citations) who ranked third working in Fac Med USP published a review titled “Clinical research with transcranial direct current stimulation (tDCS): Challenges and future directions” as the first author, and NITSCHE MA, FREGNI F, and him co-published this review in 2012 which is a highly cited paper (cited frequency = 809). Cheng et al. (2022) collaborated with BRUNONI AR published a network meta-analysis (NMA) concerning the non-invasive brain and nerve stimulation techniques for migraine prophylaxis and aiming to compare strategies of non-invasive brain/nerve stimulation for migraine prophylaxis with respect to their effectiveness and acceptability, and the study found that present NMA demonstrated that the hf-TMS-C3 and hf-tONS-Oz (the specific protocols of tDCS and rTM) were associated with the most effective way in outcomes of monthly migraine days and response rate, respectively. Also, c-tDCS-CP4 + a-tDCS-arm, in addition to significantly improving monthly migraine days, was the most effective approach among the interventions in improving migraine pain severity. As a result of the limitations of the small sample sizes, heterogeneous primary outcomes, and study design among the included RCTs and relatively short follow-up durations, this study may not be very accurate (Cheng et al., 2022).

Last but not least, as for the ranking list of both authors or co-cited authors, NITSCHE MA and FREGNI F always ranked first and second, respectively, indicating that their research is of absolute importance in the field of tDCS.

#### Bibliometric analysis of the higher-impact journals

The emergence of academic journals, which play an essential role in the presentation of research results, further scientific research, and science communication of scientists, is related to the development of economics, politics, science, and technology, as well as our demand for academic knowledge. Scientific publications appear as carriers for achievements in scientific research, and the analysis of the distribution of journal sources is helpful for researchers to quickly locate the most appropriate journals for their articles (Butt et al., 2021). The 4,756 publications related to tDCS were published by a total of 200 scholarly journals and 324 co-cited journals. The top 20 journals are shown in Table 3. The Journal Impact Factor (JIF) is a significant parameter for evaluating journals’ value, and it developed by Garfield was meant to be a measurement of a 2-year moving average citation of a journal (Garfield, 2006). The journal named *BRAIN STIMULATION* with IF, 2022 = 9.184, published the highest number of publications on tDCS (306 publications) and was cited 4,041 times, followed by *FRONT HUM NEUROSCI* (210 publications; IF, 2022 = 3.473; 2,089 citations), *PLOS ONE* (117 publications; IF, 2022 = 3.752; 2,508 citations) and *FRONTIERS IN NEUROSCIENCE* (111 publications; IF, 2022 = 5.152). Based on JIF, the Journal Citation Reports likewise divided journals from the WoS categories into four equal sections, with the top 25% being attributed to Q1, the top 25–50% to Q2, and so forth. From Table 3, we can learn that 25% of journals and 45% of co-cited journals were classified as Q1. The frequency of co-citations, which indicates whether a journal has a significant impact on a particular research field, determines the influence of journals.

The main research direction of these journals was Neurosciences, Clinical Neurology, and Psychiatry. Furthermore, *BRAIN STIMULATION* ranked No. 1 both in journal and co-cited journal, which indicated that *BRAIN STIMULATION* had an absolute influence in the field of tDCS.

### Research topic and hotspots analysis

#### Analysis of highly-cited publications

It is commonly accepted that a publication’s significance may be evaluated by the number of citations it receives, and the more frequently a publication is cited, the more popular a field is (Gao et al., 2020). The volume of global publications on tDCS is rising, and it is also expected to continue expanding in the following few years. The cumulative citation frequency of the 4,756 articles reached 177,683 times, and it reached 85,004 times excluding self-citation frequency. The average citation frequency of the articles was 37.36, and the H-index was 175. Among the 4,756 articles, the cited frequency of the top 10 articles is shown in Table 4. The publication titled “Excitability changes induced in the human motor cortex by weak transcranial direct current stimulation,” whose average annual citation frequency (144.74) and total citation frequency (3,329), respectively, ranked first was published in 2002. Followed were “Transcranial direct current stimulation: State of the art 2008” (average = 126.07, 3,329 citations) and “Sustained excitability elevations induced by transcranial DC motor cortex stimulation in humans” (average = 78.91, 1,736 citations). Furthermore, the first author of these three articles above was Nitsche MA. In addition, Nitsche MA also participated in the research of seven publications among the top 10 papers with the highest cited frequency as the first author.

#### Analysis of co-cited references

**FIGURE 8:**
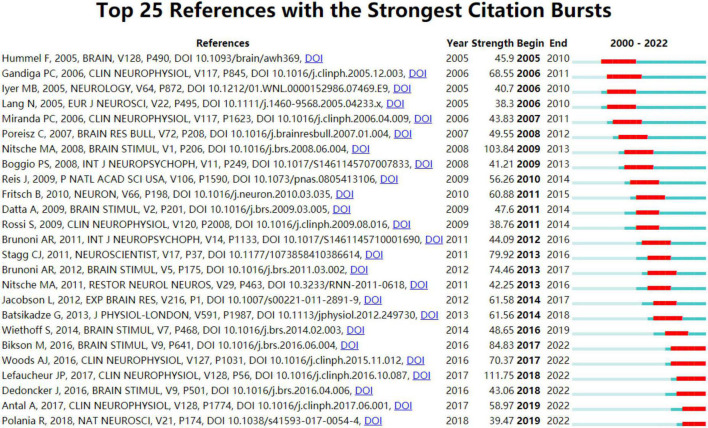
The top 25 co-cited references with the strongest citation bursts.

Co-cited references are references cited collectively in the reference lists of other literature (Lu et al., 2019). The top ten co-cited references are presented in Table 5. As shown in Figure 8, the blue lines indicated the time interval, and the red part represented the time period when the reference burst occurred. The burst strength of the top 25 references with the strongest citation bursts ranged from 38.3 to 111.75. “Lefaucheur et al. (2017)” had the highest burst strength (111.75, 2018–2022), followed by “Nitsche et al. (2008)” (103.84, 2009–2013) (mentioned in the authors and co-authors’ analysis). In addition, the burst strength of the remaining co-cited references was all less than 100. As shown in Figure 8, we learned that six of the top 25 references occurred in recent years (Bikson et al., 2016; Dedoncker et al., 2016; Woods et al., 2016; Antal et al., 2017; Polania et al., 2018; Zhong et al., 2020), and Lefaucheur et al. (2017) was one of the six references, corresponding publication titled “Evidence-based guidelines on the therapeutic use of transcranial direct current stimulation (tDCS)” whose co-cited frequency reached 337 times and ranked first of the top 10 co-cited references in Table 5, which demonstrated that the European Chapter of the International Federation of Clinical Neurophysiology commissioned a group of European experts to gather information on the state of the science frontier in the therapeutic use of tDCS from studies published until September 2016 regarding pain, Parkinson’s disease, other movement disorders, motor stroke, poststroke aphasia, epilepsy, consciousness disorders, Alzheimer’s disease, tinnitus, depressive disorders, and more. This publication was a high-cited article, and the citation frequency of the article entered the top 1% in the academic field of Neuroscience & Behavior based on the high citation threshold of the relevant area and publication year.

**TABLE 5:** The top 10 co-cited references in research of transcranial direct current stimulation (tDCS).

Ranking	Co-cited references	Frequency	Centrality	Year
1	Lefaucheur et al., 2017	337	0.12	2017
2	Bikson et al., 2016	270	0.03	2016
3	Woods et al., 2016	258	0.01	2016
4	Batsikadze et al., 2013	200	0.05	2013
5	Brunoni et al., 2012	198	0.00	2012
6	Stagg et al., 2011	183	0.01	2011
7	Jacobson et al., 2012	177	0.00	2012
8	Nitsche et al., 2008	168	0.01	2008
9	Wiethoff et al., 2014	167	0.13	2014
10	Horvath et al., 2015	157	0.66	2015

**FIGURE 9:**
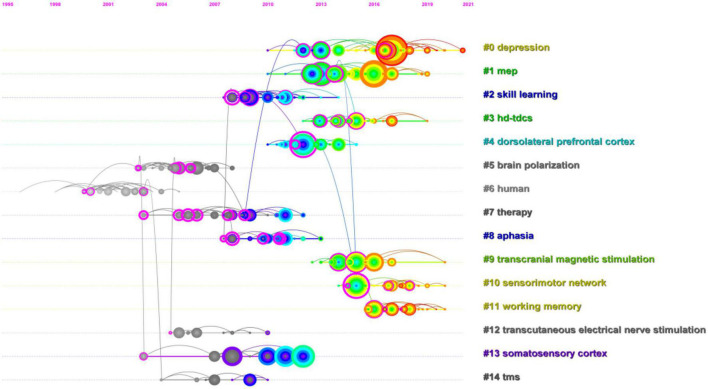
CiteSpace visualization map of reference co-citation analysis timeline viewer related to transcranial direct current stimulation (tDCS).

It was observed from Figure 9 that “depression” #0, “Sensorimotor network”#10, “working memory”#11, and “Transcranial magnetic stimulation”#9 were relatively the latest research focusing in the co-cited reference and replaced “mep”#1, “skill learning”#2, “hd-tdes”#3, “dorsolateral prefrontal cortex”#4, “brain polarization”#5, “human”#6, “therapy”#7, “aphasia”#8, “transcutaneous electrical nerve stimulation”#12, “somatosensory cortex”#13, and “tms”#14.

#### Analysis of keywords

A hot subject of a research field is represented by high-frequency keywords, and the location and significance of the associated research content in that field are shown by high-centrality keywords (Zhong et al., 2020). As shown in Table 6, “direct current stimulation” appeared 883 times and ranked first, followed by “transcranial direct current stimulation” (810), “tdc” (739) “transcranial magnetic stimulation” (694), “excitability” (668), and so on. “Prefrontal cortex,” the highest centricity among the top 20 keywords, reached 0.87, followed by “transcranial magnetic stimulation” (0.86) and “human motor cortex” (0.77). The researchers of Batista, EK, Klauss, J, Fregni, F, and Nitsche, MA demonstrated that repetitive bilateral transcranial direct current stimulation over the dorsolateral prefrontal cortex could reduce craving for crack cocaine use in order to reduce anxiety (Kaski et al., 2014).

**TABLE 6:** The top 20 keywords in research of transcranial direct current stimulation (tDCS).

Ranking	Keyword	Centricity	Count	Year
1	Direct current stimulation	0.07	883	2004
2	Transcranial direct current stimulation	0.59	810	2007
3	tdc	0.07	739	2012
4	Transcranial magnetic stimulation	0.86	694	2004
5	Excitability	0.29	668	2004
6	Brain stimulation	0.26	605	2008
7	Modulation	0.23	585	2003
8	moTor cortex	0.32	580	2007
9	Non-invasive brain stimulation	0.26	529	2011
10	Human motor cortex	0.77	477	2004
11	Prefrontal cortex	0.87	434	2007
12	Working memory	0.23	426	2008
13	Cortex	0.13	380	2009
14	Magnetic stimulation	0.61	357	2004
15	Dorsolateral prefrontal cortex	0.13	353	2010
16	Brain	0.10	314	2001
17	Double blind	0.17	261	2010
18	Plasticity	0.23	235	2004
19	Performance	0.07	233	2012
20	DC stimulation	0.26	225	2007

**FIGURE 10:**
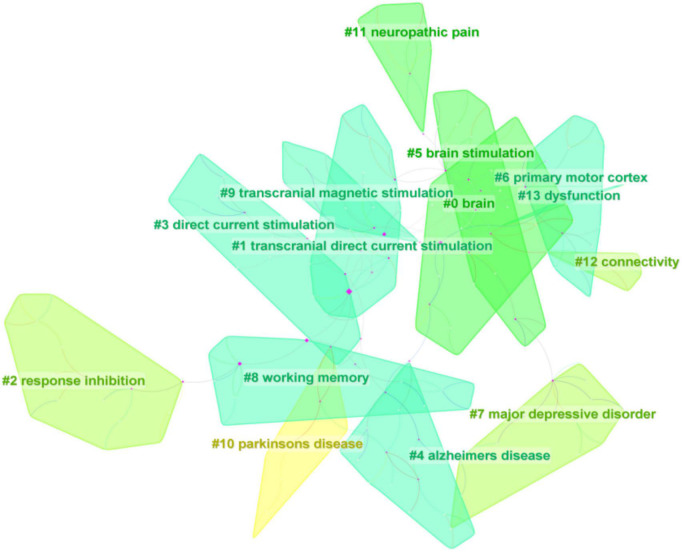
Clustering map of reference co-citation related to transcranial direct current stimulation (tDCS), 2000–2022.

**FIGURE 11:**
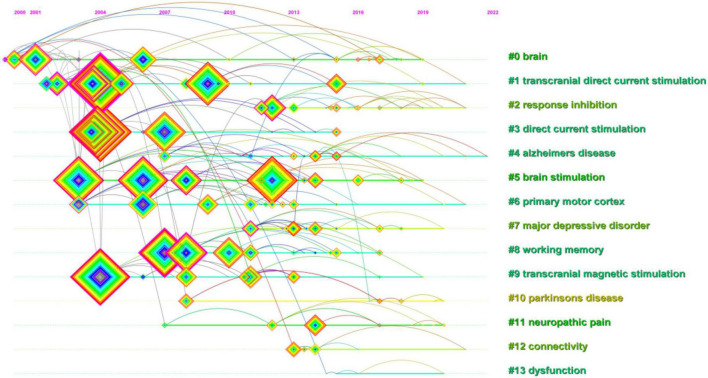
CiteSpace visualization map of keywords analysis timeline viewer related to transcranial direct current stimulation (tDCS).

**FIGURE 12:**
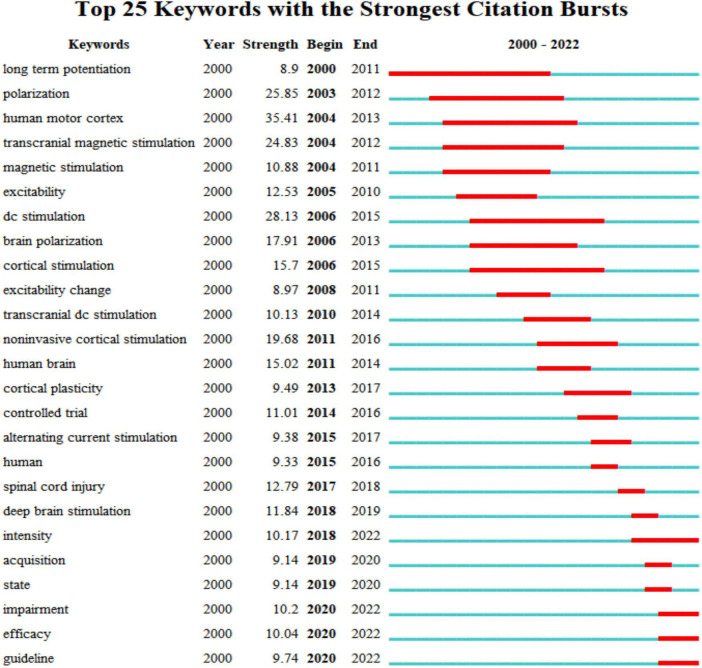
Keywords with the strongest citation bursts in publications related to transcranial direct current stimulation (tDCS).

The clusters were named by extracting nominal terms as labels from the titles of the cited articles. The LLR (log-likelihood ratio) algorithm is applied as the extraction method, and we finally gained the clustering function in Figure 10. The network map consists of 13 distinctive clusters, and nodes within the same cluster might have a similar research direction to the node from other clusters. As shown in Figure 10, “Brain”#0 was the largest cluster. Significantly, “Parkinson’s disease” was a relatively new cluster and a new research topic. In a study conducted by Kaski et al. (2014), they found that anodal transcranial direct current stimulation during physical training caused patients with Parkinson’s disease to slip gait and balance in 16 community-dwelling patients who underwent transcranial direct current stimulation and other intervention conditions. The correlation of various cortical regions for inhibiting reactionary responses is widely studied using the tDCS, followed by “response inhibition”#2, “major depressive disorder”#7, and “connectivity”#12. Currently, research on tDCS reported polarity-, time-, and stimulation-site-dependent effects on response inhibition. The dorsolateral prefrontal cortex (dlPFC), which has undergone several functional magnetic resonance imaging investigations, has been identified as a key brain region for reaction inhibition. However, its mechanism of action is yet unclear. According to the study made by Chen et al. (2021) anode and cathode tDCS of the right dlPFC improved response inhibition, with the right dlPFC perhaps playing a significant role in this process. In a clinical trial including 30 bipolar disorder (BD) patients, Mardani et al. (2021) demonstrated that pharmacological therapy using transcranial direct current stimulation (tDCS) could lessen depressive symptoms and enhance response suppression (BD is linked to depressive symptoms and impaired executive function, such as response suppression). What’s more, we also obtained the timeline view for the 13 clusters in Figure 11. “brain”#0 first appeared in 2000 and was the earliest keyword. The research focused on “dysfunction”#13 appearing latest among the 13 clusters. The timeline for “Alzheimer’s illness” presented that it was the closest to 2022, which suggested that this subject might have received more attention lately and become a research hotspot soon. Burst keywords can also be identified as indicators of emerging trends. Figure 12 presented keywords with the strongest citation bursts in this field. “Human motor cortex” broke out first in 2004 and ended in 2013 whose strength reached 35.41 ranking first among the 25 keywords. The second highest was “dc stimulation” (28.13, 2007–2013), followed by “polarization” (25.85, 2003–2012). In addition, “dc stimulation” and “polarization” were the major topic during the early stage. Significantly, “Intensity,” “impairment,” “efficacy,” and “guideline” had citation burst, indicating that these keywords earned excessive attention in recent years.

## Major findings

We systematically summarized a series of specialized terms that appear in the Section “Results and discussion,” so that scholars can have a better understanding of the relevant study of tDCS and the research direction in the future.

### The development history of transcranial direct current stimulation

According to the data in Figure 2, the growth rate of publications on tDCS can be divided into two stages: a slow phase from 2000 to 2009 and a fast growth phase from 2010 to 2021, and the growth rate of the number of articles on tDCS published from 2010 to 2011 was the highest, reaching 101.49%. It is worth mentioning that we collected literature related to tDCS from 2000 to 2022, but it doesn’t mean that the development of tDCS began in 2000. Transcranial direct current stimulation (tDCS) with a long history is a non-invasive transcranial stimulation technique that uses positive and negative electrodes to apply a slight current (1–2 mA) to specific scalp sites to regulate cortical excitability. During 43–48 AD, the (electric) torpedo fish was discovered to relieve alleviate gout and headache by causing a strong electrical discharge when it was placed on the scalp (Dolhem, 2008). From then on, scientists started to explore the application and research of electrical stimulation in medicine. In 1775, French physician Charles Le Roy (1726–1779) placed wires around the heads and legs of blind subjects so that they could sense vision (Pascual-Leone and Wagner, 2007). At the beginning of the nineteenth century, Italian doctor Giovanni Aldini (1762–1834) applied electrotherapy to patients with deafness, amaurosis, and mental illness, electrotherapy did work well (Arêas et al., 2020). In 1923, Italian scientist Alessandro Volta (1745–1827) discovered that the contraction of the frog muscle is produced by the contact of two different metals which are the generators of the bimetallic current (Dolhem, 2008). In 1956, Terzuolo and Bullock (1956) found that an electric field of 10 mV/cm was sufficient to regulate the spontaneous firing of crayfish neurons. From 1960 to 1998, extensive clinical studies on tDCS were carried out, and a large amount of evidence showed that depression could be improved by tDCS. In 2000, Nitsche and Paulus (2000) proved that tDCS can change the excitability of the human motor cortex. Thus, the clinical research of tDCS opened a prelude. Fregni et al. (2005) found that tDCS can improve working memory in 2005, and Zaehle et al. (2011) found electrophysiological evidence that tDCS does improve working memory in 2011. In 2013, all these results suggested that an enhancement of tDCS intensity not only necessarily increased the efficacy of stimulation but might also shift the direction of excitability alterations (Batsikadze et al., 2013). In 2017, Lefaucheur et al. (2017) with a comprehensive evidence-based analysis of the reported clinical efficacy of tDCS for the first time could lead to therapeutic applications in the neurological, otorhinolaryngological, and psychiatric domains. Compared with tDCS, TMS is also a typical non-invasive transcranial stimulation technique that is receiving extensive attention and in-depth research due to its advantages in the treatment of brain function, non-invasive, easy operation, and low cost. Meanwhile, common adverse reactions such as itching, headache, and burning are usually mild and have no long-term effects (Jonker et al., 2021).

### Functional mechanisms of transcranial direct current stimulation

The stimulation mode of tDCS includes three stimulation modes: excitatory stimulation, inhibitory stimulation, and spurious stimulation. Furthermore, the stimulation parameters include stimulation site, current intensity, stimulation duration, authentic stimulation, and spurious stimulation. The development of tDCS dates back hundreds of years, and it is widely used in the study of various neuropsychiatric diseases, but its mechanism of action is still unclear up to now. Based on the current state of research, we will explain the mechanism of tDCS from the following three aspects.

#### Cortex excitability

In research on tDCS, “excitability” ranked 5th among the top 20 keywords (Table 6), with centrality (0.29) and publications (668). The current from the anode to the cathode electrode creates a weak electric field throughout the cortex in the condition of tDCS stimulation. In general, cerebral excitability can be increased by anodal stimulation causing neuronal depolarization, while cathodal stimulation is the contrary (Nitsche and Paulus, 2000). However, this effect has been found to be highly variable currently. Jonker et al. (2021) designed a large double-blind placebo-controlled trial: in a pre-registered, double-blind, randomized, and placebo-controlled experiment with repeated measures, cortical excitability over the left motor cortex of 62 healthy volunteers was assessed both before and after anodal tDCS at 2 mA for 20 min. This research suggested that cortical excitability may not be reliably affected by anodal tDCS at 2 mA for 20 min (Jonker et al., 2021). It was reported that tDCS is a promising technique for consciousness improvement for patients with DOC (disorders of consciousness). Nevertheless, without direct electrophysiological proof to indicate the impact of tDCS on individuals with DOC, Bai et al. (2017) used TMS-EEG to measure the changes in cortical excitability (Table 6, ranking = 15, centricity = 13, count = 353) after 20 min of anodal tDCS to the left dorsolateral prefrontal cortex. The results revealed that tDCS can successfully help patients with DOC by regulating cortical excitability (Bai et al., 2017). The threshold of action potentials can be altered using tDCS by changing the polarity of the neuronal membrane. Usually, the fact that tDCS produces an electric field is a subthreshold stimulus, which suggested that the tDCS itself does not result in neuronal depolarization (Fritsch et al., 2010).

#### Synaptic plasticity

The changes in synaptic plasticity are often characterized by long-term potentiation (LTP) and long-term depression (LTD) of synaptic activity. Direct current stimulation applied to cortical neurons regulates the expression of N-methyl-D-aspartate receptor and the release of γ-aminobutyric acid, resulting in long-term strong or long-term inhibitory effects and synaptic remodeling (Fritsch et al., 2010). In addition, there is metaplasticity, the modification of plasticity induction, including direction, magnitude, and duration of previous activity of the same postsynaptic neuron or neuronal network. Furthermore, it is a more advanced form of plasticity, which means that the activity of the cell or synapse before will affect the direction or degree of synaptic plasticity, and the change in synaptic plasticity will accordingly alter the activity of the cell (Müller-Dahlhaus and Ziemann, 2015). Research showed that tDCS with spacers has effects on motor plasticity, and these effects may be described based on metaplasticity. By studying the effect of different inter-stimulation intervals on the performance of a three-back task, Carvalho et al. (2015) tested several tDCS-based metaplasticity protocols in working memory (WM). The outcome demonstrated that the performance in the three-back task enhanced (*p* = 0.042) with a 10-min interval between two cathodal tDCS sessions. The findings showed that the polarity effects of tDCS on working memory rely on the previous degree of activity of the recruited neural population (Carvalho et al., 2015). In Table 6, “plasticity” ranked 18th with centricity (0.23) and count (235). In addition, it was revealed from Figure 9 that through visualization map of reference co-citation analysis timeline viewer “working memory”#11 had more occurrence in co-cited references, and “working memory”#8 was also a relatively large cluster in Figure 10, which indicated that it is a research hotspot in recent years.

#### Functional connectivity

“Connectivity” was the 12th in Figure 11, and it recently attracted a lot of attention. A wide variety of illnesses involving abnormalities in the central and peripheral nervous systems are included in neurological disorders. It has been shown that individuals with neurological illnesses exhibit abnormal resting-state functional connectivity (rsFC), which is linked to patients with ongoing functional impairment. The tDCS has recently been demonstrated to enhance rsFC, although the outcomes were conflicting (Chan and Han, 2020). According to studies made by Chan and Han (2020) both localized (i.e., brain regions under the transcranial electrodes) and diffused (i.e., brain regions not directly impacted by the transcranial electrodes) rsFC can be altered by active tDCS.

### Research and application of transcranial direct current stimulation

The application of tDCS technology in the field of neurological rehabilitation has been gradually promoted in this century. Current research showed that tDCS has different therapeutic effects on post-stroke hemiplegia, cognitive disorders, speech and swallowing disorders, depression, acute mania, bipolar disorder, panic, hallucinations, obsessions/compulsions, schizophrenia, catatonia, posttraumatic stress disorder, drug cravings, and neurologic diseases such as Parkinson’s disease, dystonia, tics, stuttering, tinnitus, spasticity, and epilepsy, and pain syndromes such as neuropathic pain, visceral pain, and migraines (Lefaucheur, 2016). In Figure 9, “depression”#0 ranked first in the timeline of the reference co-citation analysis and was the latest cluster. Nowadays, the method for using tDCS for major depressive disorder involves either increasing neural activity in the left dorsolateral prefrontal cortex (DLPFC) with anodal stimulation and/or decreasing neural activity in the right DLPFC with cathodal stimulation (Brunoni et al., 2011). In the recommendations for appropriate clinical practice, tDCS is acknowledged with level B evidence on the antidepressant effectiveness of anodal tDCS of the left DLPFC (Mutz et al., 2018). In terms of the therapeutic effects, current evidence demonstrated that tDCS has limited antidepressant benefits in those with treatment-resistant (Bennabi and Haffen, 2018), so it still remains a viable avenue. Generally, tDCS is an intriguing approach for treating children and adolescents for the same disorders compared with adults due to its non-invasive nature and common absence of adverse effects (Buchanan et al., 2019). At the same time, the application of NIBS is not limited to clinical situations. Thus, patients may consider it more convenient to receive their treatment remotely while lounging at their own homes. In addition, several therapy trials and various guidelines for remotely supervised and at-home tDCS have been accomplished (Buchanan et al., 2020), which indicated that tDCS has a good development prospect in the field of depression and is a hot research direction at present. Furthermore, “Alzheimer’s disease” #4, ranking 5th in the map of the keywords analysis timeline in Figure 11, was also the latest cluster, which suggested that the research and application of tDCS in Alzheimer’s disease may become a new researchful direction and research hotspot in recent years. The most common method of transcranial electrical stimulation used to treat Alzheimer disease (AD) is tDCS. Studies on the application of tDCS for the treatment of AD have focused on the left DLPFC, left temporal lobe, and temporoparietal lobe (Lefaucheur, 2016). According to research by Saxena and Pal (2021), brain stimulation techniques are a relatively new breakthrough in slowing the course of AD. Commonly, three different kinds of brain stimulation methods used currently are repetitive transcranial magnetic stimulation, transcranial direct current stimulation (tDCS), and deep brain stimulation (DBS). Among three of them, we believed that tDCS is particularly essential for the treatment of AD because of the portability of the device, low cost, and easy to use. In addition, treatment using tDCS is very convenient and doesn’t disrupt daily activities. Due to the tDCS with only low-intensity current, it may be performed as an outpatient procedure without the need for anesthesia, which is essential for developing countries with low healthcare budgets and where more percentage of patients depend on daily wages. Patients should be more compliant with the treatment because it can be done in a relatively shorter time span (Saxena and Pal, 2021).

In a nutshell, the present difficulty with non-pharmacological treatments is standardizing the protocol’s settings, and a potential future strategy is to develop different standardized protocols depending on the severity of the illness (Yu et al., 2021).

## Strengths and limitations

British intelligence scientist Pritchard made the initial proposal for bibliometrics (Pritchard, 1969). Bibliometrics is a quantitative analytic technique based on a variety of features of literature, such as the number and year of publications, authors, countries, institutions, and keywords, which take advantage of mathematical and statistical ways to show the status and trend in a certain area (Zhu and Meng, 2013). However, as far as we know, this was the first-ever study of peer-reviewed publications relative to tDCS using several scientometric and visual analytic methods, which is one of the biggest innovations of this study at the same time. Moreover, to fully assess the current status of research on tDCS, two main visualization tools were utilized together. Firstly, citation, co-citation, and co-occurrence analyses between countries, institutions, authors, journals, publications, references, and keywords were analyzed and visualized using CiteSpace. Secondly, bibliometric features including the number of publications and citations, citation frequency, H-index, journal’s impact factors, and journal citation reports were summarized using Microsoft Office Excel. In addition, publications on tDCS from 2000 to 2022 on tDCS were extracted from the Web of Science Core Collection (WOSCC) database as a data source using CiteSpace and Microsoft Excel to analyze and visualize. Through the bibliometric method, we gained an in-depth understanding of the current research status and development trend on tDCS through the knowledge map which quantitatively and qualitatively showed the current research status and trends in the field of tDCS. Finally, to introduce the research in this field in a more comprehensive way, we conducted a systematic analysis from the perspective of the historical development, functional mechanism, and research and application of tDCS in order to provide readers with a better vision, which was also our innovation.

This study was subject to some limitations related to bibliometrics as well. Firstly, the dataset was incomplete since it was only from the WOSCC database and neglected the other large databases leaving out a few related studies. However, it is widely believed that the WOSCC is the most commonly used reference database for bibliometric analysis (Yeung et al., 2020). Additionally, data from the WOSCC were large enough to reveal the current state of research on tDCS. Moreover, different kinds of databases have distinct features including output formats of files and count of citations, so mixing the databases together may not be the best option. Secondly, the contributions from non-English speaking nations may be underestimated since we only chose publications in English and omitted publications in other languages. Thirdly, due to the ongoing updating of the literature in the WOSCC, the impact of recently published high-quality articles may also be underestimated for they may not receive enough citations, which indicated that there was a difference between the retrieval results of this study and the actual number of publications.

Moreover, CiteSpace still has several limitations that need to be resolved before it can fully replace system retrieval. Firstly, the quality of the extracted publications varied, which may weaken the validity of the analysis. Secondly, due to the incomplete keyword extraction, several core keywords were partly included in the study. However, our research and analysis results might provide worthwhile information and some reference and lay a foundation for academic researchers and clinicians to clearly gain an in-depth understanding of the current research status and development trend on tDCS.

## Conclusion

In conclusion, we calculated and assessed the information of publications with regard to different countries, institutions, authors, co-author, journals, etc., by CiteSpace (6.1.R3) at first, and then analyzed the topics and hotspots to predict the research trends on tDCS. Clinical applications of tDCS, such as neurologic diseases, post-stroke, pain syndromes, and psychiatric conditions, have been intensively studied in the past few years. Among them, studies on reactive response inhibition, depression, Alzheimer’s disease, Parkinson’s disease, and functional connectivity in the mechanism of tDCS will certainly become the focus of research.

## Data availability statement

The original contributions presented in this study are included in the article/supplementary material, further inquiries can be directed to the corresponding authors.

## Author contributions

WS and XC designed the research subject. JS and XK conducted the literature retrieval and screening. XD, ZL, and BH provided guidance in statistical analysis. WS, JS, and WZ wrote the manuscript. XC and ZF critically revised the manuscript. All authors read and approved the final manuscript.
